# Genomic and Epigenomic Biomarkers of Immune Checkpoint Immunotherapy Response in Melanoma: Current and Future Perspectives

**DOI:** 10.3390/ijms25137252

**Published:** 2024-06-30

**Authors:** Sultana Mehbuba Hossain, Carien Carpenter, Michael R. Eccles

**Affiliations:** 1 Department of Pathology, Dunedin School of Medicine, University of Otago, Dunedin 9016, New Zealand; mehbuba.hossain@otago.ac.nz (S.M.H.); carca489@student.otago.ac.nz (C.C.); 2 Maurice Wilkins Centre for Molecular Biodiscovery, Level 2, 3A Symonds Street, Auckland 1010, New Zealand

**Keywords:** melanoma, immunotherapy, PD-L1 expression, mutation burden, neoantigens, DNA methylation, m6A RNA methylation, long non-coding RNAs

## Abstract

Immune checkpoint inhibitors (ICIs) demonstrate durable responses, long-term survival benefits, and improved outcomes in cancer patients compared to chemotherapy. However, the majority of cancer patients do not respond to ICIs, and a high proportion of those patients who do respond to ICI therapy develop innate or acquired resistance to ICIs, limiting their clinical utility. The most studied predictive tissue biomarkers for ICI response are PD-L1 immunohistochemical expression, DNA mismatch repair deficiency, and tumour mutation burden, although these are weak predictors of ICI response. The identification of better predictive biomarkers remains an important goal to improve the identification of patients who would benefit from ICIs. Here, we review established and emerging biomarkers of ICI response, focusing on epigenomic and genomic alterations in cancer patients, which have the potential to help guide single-agent ICI immunotherapy or ICI immunotherapy in combination with other ICI immunotherapies or agents. We briefly review the current status of ICI response biomarkers, including investigational biomarkers, and we present insights into several emerging and promising epigenomic biomarker candidates, including current knowledge gaps in the context of ICI immunotherapy response in melanoma patients.

## 1. Introduction

Firstline therapy for cancer patients involving immune checkpoint inhibitors (ICIs) is now the standard of care for several late-stage cancers, such as melanoma, colorectal cancer, head and neck cancers, as well as non-small cell lung cancer (NSCLC) [1,2,3]. Generally, ICI therapies disrupt cancer immune tolerance through immune regulatory checkpoints and strengthen the anti-tumour immune response [1]. Ipilimumab (an anti-CTLA-4 antibody), Nivolumab and Pembrolizumab (both anti-PD-1 antibodies), and Atezolizumab (an anti-PD-L1 antibody) act by targeting specific immunosuppressive checkpoints [4]. Ipilimumab targets cytotoxic T lymphocyte antigen 4 (CTLA-4) on T cells, while Nivolumab and Pembrolizumab target programmed cell death protein 1 (PD-1) on T cells, and Atezolizumab targets programmed cell death ligand 1 (PD-L1) on tumour cells and tumour-infiltrating immune cells [4]. T cells can be divided into two main groups: CD4+ T cells, which are highly versatile and polyfunctional, and CD8+ cytotoxic T lymphocytes (CTLs). CTLA-4 is an inhibitory protein receptor expressed by both CD4+ and CD8+ T cells that directly competes with CD28 for the ligands CD80 and CD86 and interrupts T-cell priming, leading to immunosuppression [5,6,7,8]. The binding of anti-PD1 or anti-PD-L1 antibodies to PD-1 or PD-L1, respectively, prevents the interaction between PD-1 and PD-L1 and results in the prolonged activation of T-cell responses, including potent tumour-specific immune responses [9].

Anti-PD-1 therapy in melanoma promotes the increased presence of tumour-infiltrating lymphocytes (TILs), as well as restoring functionality in exhausted T cells [9]. However, cancer immunotherapy has several limitations, including an inability to predict the efficacy or response to treatment, the development of cancer immunotherapy resistance, inadequate measures to reduce toxicity, and overall high treatment costs [10]. Nevertheless, combining immune checkpoint (e.g., CTLA-4 and PD-1) blockers has a synergistic effect in increasing the patient response by activating anti-tumour immune responses in dual pathways. Clinical data show that 20–40% of melanoma patients respond to these monotherapies, whereas around 60% of patients respond to treatment with a combination of CTLA4 and PD-1 blockers [11]. Anti-CTLA-4 activates Treg cells (Tregs), which suppress dendritic cells (DCs) in lymph nodes, and simultaneous anti-PD-1 treatment inhibits effector T cell (Teff) and natural killer cell (NK) activation in peripheral tissues, inducing regulatory T cell (Treg) differentiation, meanwhile facilitating anti-tumour response rates. The combination of Ipilimumab and Nivolumab therapy has accordingly been approved for the treatment of melanoma and several other cancers, including tumours with microsatellite instability [12].

Diagnostic, prognostic, and predictive biomarkers are essential tools used in the clinical management of melanoma patients. Diagnostic biomarkers such as fluorescence in situ hybridization (FISH), comparative genomic hybridization (CGH), and myPath (Myriad Genetics, Salt Lake City, UT, USA) are used to assist in melanoma diagnosis [13]. Prognostic biomarkers help to estimate whether the tumour is likely to progress or remain indolent in the absence of treatment. Predictive biomarkers help to predict how well a patient will respond to treatment [14].

One important predictive biomarker, known as PD-L1, CD274, or B7–H1, is a transmembrane protein that is frequently expressed on tumour cells. PD-L1 interacts with the PD-1 receptor on T cells, leading to host immune system evasion [7]. However, as a single biomarker, PD-L1 expression is imperfect and has marked limitations in predicting the response to ICI anti-PD1/PD-L1 therapy [15]. The expression of PD-L1 on at least 50% of tumour cells in immunohistochemistry (IHC) on formalin-fixed, paraffin-embedded (FFPE) tissue sections is a mandatory test used in some medical centres for prescribing Pembrolizumab as first-line monotherapy in NSCLC [16].

Additional investigations on PD-L1 expression and its role in tumour biology were reviewed elsewhere [17,18,19,20]. As a PD-1 blockade is dependent on T-cell recognition of tumour antigens, it may prove ineffective in cases where T cells lack TCRs corresponding to tumour antigens, where tumours fail to present antigens via their MHC, or where there is a lack of tumour-infiltrating lymphocytes (TILs) [21]. Furthermore, focal PD-L1 expression in IHC may sometimes be overlooked in small biopsy samples such as needle biopsies. PD-L1 expression can vary among different tumour lesions in the same patient over time and depending on the location. Additionally, PD-L1 can be expressed by various cell types within the tumour microenvironment, complicating the scoring and interpretation process [22]; therefore, it has been recommended that PD-L1 expression should not be used to guide the choice of combined (anti-CTLA-4 and anti-PD-1) ICI therapy for patients [23].

To improve the prediction of patient responses to ICI treatment, additional information regarding indicators or markers in patients who respond positively is required. Molecular biomarkers are considered a powerful tool for the prediction of treatment response in patients, because they potentially correlate strongly with pathological changes occurring in cells [23,24]. Identifying effective biomarkers for metastatic melanoma immunotherapy has become a primary challenge and remains a critical priority to optimize personalized medication with ICI therapy for patients who are responsive to treatment, while patients not responsive to ICI treatment could proceed with other therapies to receive significant treatment outcomes, avoiding severe side effects and minimizing treatment costs [11]. A recent study conducted a systematic review on the most recent findings on the development or validation of prognostic biomarkers in malignant melanoma treatments [25]. In the present review, we are focused exclusively on cutaneous melanoma tissue-based biomarker studies related to ICI therapy.

In the following sections we discuss the genomic biomarkers associated with the response to ICI therapy, after which we discuss the growing field of epigenomic biomarkers of response to ICI therapy, including DNA and RNA methylation and non-coding RNAs, and how these could impact patient outcomes associated with ICI therapy (Table 1).

## 2. Genomic Biomarkers of ICI Treatment Response in Melanoma Patients

Genomic characteristics that are associated with an improved ICI response in cancer patients help to distinguish biomarkers for an improved prediction of the response to immunotherapy. In this section, we discuss three widely studied genomic biomarkers associated with ICI response: tumour mutational burden, neoantigen expression, and mismatch repair deficiency/high microsatellite instability.

### 2.1. Tumour Mutational Burden (TMB)

ICI therapy exhibits a higher efficacy in tumours with enriched clonal genetic abnormalities and a higher mutation burden, which suggests that tumour mutational burden (TMB) acts as a potential biomarker for predicting responses to ICIs [37]. TMB is defined as the total number of somatic mutations per megabase of DNA or of non-synonymous mutations in tumour tissues, including replacement and insertion–deletion mutations [38]. The relevance of this biomarker is based on the hypothesis that an elevated number of exonic mutations in tumours leads to an increase in neoantigen production, which could then be recognized by CD8+ T cells, resulting in improved immune responses [26,27]. This phenomenon is thought to be evident in melanoma, where high levels of UV-induced mutations are thought to lead to an increased level of tumour neoantigens, thereby contributing to a higher immunogenic tumour microenvironment [39].

However, TMB is limited as a predictive biomarker for differentiating a complete or partial response from a progressive disease. The interplay between the T-cell response and neoantigens generated from clonal mutations [40] and the copy number alterations (CNAs) [41,42] significantly influences this limitation. For instance, driver mutations and CNAs can activate or suppress pathways that interact with tumour–immune signalling channels [43] such as the IFN-γ signalling pathway, resulting in acquired resistance to anti-PD1 therapy in metastatic melanoma [44].

Miao et al. (2018) [42] correlated gene-specific mutations and response or resistance to ICI therapy within a cohort of 249 ICI therapy-treated patients with multiple cancer types, revealing an association between *KRAS* and *EGFR* mutation statuses. This study demonstrated that a response to immunotherapy is influenced not only by mutational burden but also by mutational signature and mutational clonality [42]. Furthermore, immune activity against cancer cells depends on various factors, including mutations in the transporter associated with antigen processing (TAP) protein or in β-2-microglobulin, which may affect optimal peptide presentation to HLA class I molecules, thereby reducing the efficacy of ICI therapy despite a high TMB [40].

Additionally, an HLA-I genotype with two alleles with divergent sequences can enable the presentation of an increased diversity of neoantigens, suggesting that the HLA type also influences ICI efficacy [45]. A dominant mutational signature (such as dMMR) could be responsible for enhanced intra-tumoural heterogeneity, as it generates a large proportion of the mutational burden. Thus, the TMB itself might not directly mediate the ICI response, but may serve as a proxy for an underlying biological process that increases tumour immunogenicity and promotes the accumulation of somatic mutations [42].

Although associations between a high TMB and a response to ICIs have been reported in various cancer types, some studies fail to demonstrate a clear correlation between the TMB and ICI response. Therefore, further studies focusing on defining a predictive TMB cutoff, establishing sequencing strategies for comparable TMB detection across different laboratories, and exploring combinations of TMB with other potential markers are necessary to facilitate its routine clinical implementation.

### 2.2. Neoantigen Expression

The presence of somatic mutations in metastatic melanoma is widely acknowledged. These mutations, particularly if they are nonsynonymous mutations, result in amino acid sequence changes in proteins, leading to the generation of neoantigens. Thus, tumours with higher TMBs carry a larger number of neoantigens that theoretically increase tumour immunogenicity, thereby improving the likelihood of patient response and survival following treatment with ICI therapy [27,46,47].

Neoantigen formation begins when polypeptides are transported into the endoplasmic reticulum (ER) via a TAP complex. These peptides then bind to major histocompatibility complex (MHC) class I molecules with different affinities in the ER, and finally, the peptide-MHC class I complex gets recognized by CD8+ cytotoxic T cells, initiating the host anti-tumour immune response [30]. Various factors such as somatic mutations, alternative splicing, fusion genes, non-coding RNAs, and circular RNAs can produce tumour-specific antigen polypeptides, which, when mutated, become highly immunogenic and are expressed in malignant tumour cells [48,49].

However, the efficacy of treatment for patients with a high mutational load also depends on the subsequent recruitment of T cells into the tumour microenvironment (TME) [50]. While a high TMB increases the likelihood of tumour-specific neoantigen formation [39] and augments the number of TILs [30], it is important to note that the TMB alone (frequency per non-synonymous mutation) is not equivalent to the presence of neoantigens [27], since many oncogenic mutations do not give rise to neoantigens, underscoring the significance of neoantigen prediction as a distinct biomarker [29].

Nevertheless, the relationship between the neoantigen load and clinical outcomes from ICI therapy is not consistent among multiple cancer types. This inconsistency may arise because certain gene expression changes or genomic alterations, such as the upregulation of immune checkpoints [51], loss of HLA haplotypes [52], and somatic mutations in HLA or *JAK1*/*JAK2* genes, which reduce neoantigen presentation [44] during ICI therapy, can lead to potential changes in the neoantigen load and ultimately contribute to resistance to anti-PD1 therapy [53].

### 2.3. Mismatch Repair Deficiency (dMMR) and High Microsatellite Instability (MSI-H)

Microsatellite instability (MSI) is a rare event in most solid tumour types, except colorectal and endometrial carcinomas. MSI is characterized by variability in repetitive DNA sequences known as microsatellites and is caused by the inactivation of genes involved in the DNA mismatch repair (MMR) pathway, either through germline or somatic mutations [54]. The MMR system is crucial for maintaining DNA integrity by repairing base mismatches and other DNA errors [55]. Mismatch repair is necessary when nucleotide bases are mis-incorporated, leading to non-complementary base pairs, or when chemically damaged nucleotides are incorporated opposite undamaged ones, causing sequence misalignment.

The MMR system comprises DNA mismatch repair enzymes and four key genes: methylguanine methyltransferase (*MLH1*), postmeiotic segregation increased 2 (*PMS2*), mutS homologue 2 (*MSH2*), and mutS homolog 6 (*MSH6*) [56]. Errors detected by the MMR system may occur through strand slippage or the formation of secondary structures within repetitive sequences during replication, recombination, or repair [57]. Generally, MMR detects and repairs these anomalies and activates damage signalling pathways to initiate cell cycle arrest and apoptosis when the DNA damage is irreparable [58].

Defects in MMR are associated with an increase in mismatch errors and are responsible for MSI [59]. Microsatellite sequences, abundant and heterogeneous throughout the genome, are valuable for gene mapping and allele discrimination analyses due to their distinctive lengths [60]. MSI is associated with sporadic colorectal cancer and can result from the hypermethylation of the promoter region CpG islands in the DNA repair gene, *MLH1*, leading to its epigenetic silencing [60]. Thus, sporadic MMR-deficient tumours may be caused by either somatic mutation or epigenetic silencing of *MLH1* [61,62].

Tumours exhibiting mismatch repair deficiency (dMMR), whether due to an inherited or sporadic mutation, have a defect in one of the MMR genes (*MLH1*, *PMS2*, *MSH2*, or *MSH6*), resulting in the failure to repair errors in DNA replication. These errors are particularly prevalent in regions of repetitive DNA sequences known as microsatellites, resulting in high levels of MSI (MSI-H) [31]. MMR deficiency results in a 10- to 100-fold increase in somatic mutations [63,64], which in turn results in a high TMB and elevated tumour neoantigens [37,65]. This promotes the release of proinflammatory cytokines, eliciting the recruitment and activity of cytotoxic T cells [66,67].

**Figure 1:**
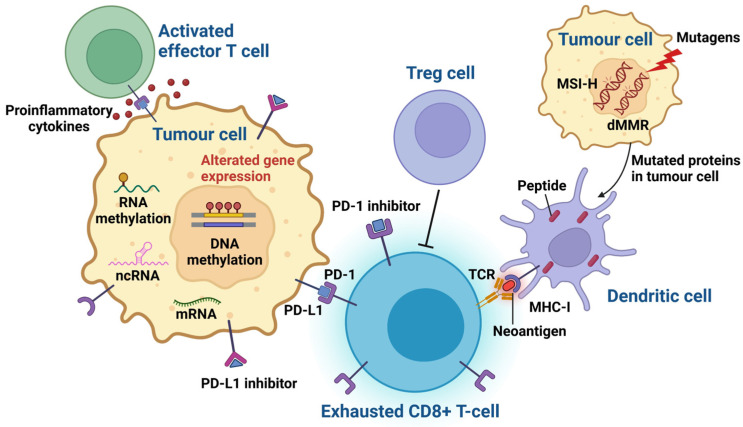
Depiction of epigenomic and genomic features in tumour cells that may impact the response to ICI therapy. As a result of exposure to mutagens, tumour cells generate mutations, including dMMR/MSI-H, which are incorporated into proteins and cause neoantigen production. Dendritic cells capture these neoantigens and activate naïve T cells through the presentation of neoantigens onto the major histocompatibility complex (MHC) and the subsequent binding with T-cell receptors (TCRs). Tumour cells frequently express PD-L1, which inhibits the immune activity by binding to PD-1. Tumour cells may also undergo epigenetic changes, including DNA and RNA methylation, which can influence non-coding RNA (ncRNA) and mRNA expression, leading to innate or acquired resistance to ICI therapy. Regulatory T cells (Tregs) may also inhibit T-cell activity and lead to “exhausted” effector T-cell (Teff) phenotypes. PD-1 inhibitors and PD-L1 inhibitors enhance the anti-tumour immune response by interrupting binding between tumour cell PD-L1 ligands and T-cell PD-1 receptors. This image was generated using BioRender.com (accessed on 1 April 2024).

Moreover, tumours with MSI-H/dMMR are associated with higher levels of CD8+ tumour-infiltrating lymphocytes, and they express higher levels of immune checkpoint proteins, including PD-1, PD-L1, CTLA-4, and LAG3, compared to microsatellite stable tumours [31]. MSI-H and dMMR are often used interchangeably due to a high consistency (90–95%) between dMMR and MSI-H in many tumours [68]. In this context, the FDA accelerated the approval of Pembrolizumab in 2017 as a second- or first-line treatment option for patients with unresectable or metastatic dMMR/MSI-H-positive solid tumours, irrespective of the tumour type or site [56]. Nivolumab is also approved for patients with dMMR/MSI-H metastatic colorectal cancer [69]. (see Figure 1).

## 3. Epigenomic Biomarkers with the Potential to Predict ICI Therapy Response

Emerging evidence suggests that epigenetic regulation plays a central role in tumour immunosurveillance, including tumour antigen production, the interaction between tumour cells and immune cells, and T-cell development, priming, activation, and exhaustion. On the other hand, tumours commonly hijack various epigenetic mechanisms to evade immune detection [70], therefore highlighting the potential for manipulating or modulating epigenetic regulators to normalize impaired immunosurveillance and/or induce anti-tumour immune responses.

Epigenetic modifications often produce stable changes in gene expression without disrupting the DNA sequence, and they can remain preserved after cell division [71]. Epigenetic modifications include DNA or RNA methylation, post-translational modifications of histone proteins, and altered chromatin remodelling, as well as non-coding RNA (ncRNA) or microRNA (miRNA) expression, which can interact at all stages of cancer development and cancer progression [71]. In addition to the comprehensive modifications observed in the tumour cell epigenome, the reconfiguration of the TME and the tumour-driven rewiring of immune cell chromatin landscapes play pivotal roles in modulating the magnitude and efficacy of the anti-tumour immune response. These alterations can substantially impact the potential response of a patient to immunotherapy and ultimately influence the overall disease outcome [72].

The long-term maintenance of transcription factor accessibility to gene regulatory elements is partly regulated by covalent modifications to histones and DNA which, in turn, affects the chromatin structure, resulting in an “epigenetic memory” of gene expression programs in dividing cell populations [73]. A recent study demonstrated that T-cell exhaustion is associated with a general increase in chromatin accessibility, with many accessible regions retained after PD-1 blockade therapy [74]. Additionally, several studies demonstrated that changes in epigenetic programming are coupled to transcriptional reprogramming during CD8+ T-cell effector and memory differentiation [73,75]. However, it remains unclear whether these reprogramming events play a direct role in regulating the effector properties in functional and exhausted CD8+ T cells. Ghoneim et al. (2017) demonstrated that progressive genome-wide de novo DNA methylation programming is critical for establishing T-cell exhaustion. Such DNA-methylation programming reinforces the repression of key genes involved in the effector function, proliferation, metabolic activity, and tissue homing in exhausted T cells. This study also revealed that these long-lived, exhaustion-associated epigenetic programs serve as a major cell-intrinsic barrier limiting the rejuvenation of antigen-specific CD8+ T cells during anti-PD1 therapy, highlighting epigenetic programs among exhausted T cells as a potential mechanism to cause anti-PD1 therapeutic failures [73]. Furthermore, DNA methylation enzymes, such as DNMT1 and DNMT3B, are upregulated in exhausted CD8+ T cells, and DNMT3A-mediated genome-wide de novo methylation can promote terminal exhaustion [73]. However, it is important to note that most studies to date describe biomarkers in bulk tissue preparations, and further investigation is required to elucidate biomarkers at the cellular level.

## 4. Understanding the Tumour Microenvironment from an Epigenetics Perspective

Carcinogenesis is a multifaceted process driven by genetic and/or epigenetic alterations within specific cells, as well as by the microenvironment in which the cells reside. The TME comprises an extracellular matrix enriched in stromal and immune cells embedded within a network of cytokines and chemokines. It has significance as a reactive platform, composing various aspects of tumour initiation, progression, metastatic spread, altered immune response, therapeutic resistance, and cancer recurrence.

At the cellular level, the process of carcinogenesis involves evading T cell-mediated immune surveillance by creating an immune-suppressive environment. Briefly, dendritic cells (DCs) capture the human leukocyte antigens (HLAs) generated by cancer cells and present them on major histocompatibility complex (MHC, MHCI, and MHCII) molecules to antigen-presenting cells (APCs). This causes the priming and activation of effector T cells (Teffs), while the regulatory T cells (Tregs) can regulate an immune response against tumour cells [76,77]. CD4+ T cells secrete different tumouricidal cytokines, such as interferon-γ (IFNγ) and TNFα to support CTLs in the disruption of primary tumour cells [78]. Epigenetic mechanisms such as DNA methylation are essential for CTL differentiation, with the transition from naïve CTLs to effector cells requiring a shift from the methylated to the demethylated states of biologically relevant gene promoters, enhancing the anti-tumoural effects [79].

Tumour-associated macrophages (TAMs) within the malignant stroma are a critical immune cell subpopulation responsible for cancer-associated inflammation, matrix remodelling, tumour immune escape, growth, invasion, angiogenesis, metastasis, cancer cell stemness, and drug resistance. Macrophage polarization between M1 (pro-inflammatory and tumouricidal) and M2 (anti-inflammatory and protumourigenic) phenotypes is regulated by distinct TMEs [80]. Pro-inflammatory CD16+ macrophages (M1) release T-cell recruiting chemokines, interact with anti-CTLA-4 antibodies, and have been associated with a positive response to combination immune therapy (anti-PD-1 and anti-CTLA-4) [81,82]. Moreover, M1 macrophages, which are PD-L1+ and localized close to cytotoxic T cells, are highly correlated to ICI-responsive patients [83]. Additionally, M1 macrophages are enriched in melanocytic “hot” melanomas, whereas M2 macrophages are enriched in neural crest-like “cold” melanomas [84], where “hot” versus “cold” tumours are characterized by a high infiltration of TILs. Notably, epigenetic regulation also influences macrophage activation and polarization, with DNMT3B knockdown promoting an alternatively activated M2 phenotype and DNMT3B overexpression acting as a negative regulator of M2 macrophage polarization [85]. Ishii et al. [86] reported that chromatin remodelling is mechanistically important in the acquisition of the M2-macrophage phenotype. M2-macrophage marker genes are epigenetically regulated by reciprocal changes in histone H3 lysine-4 (H3K4) and histone H3 lysine-27 (H3K27) methylation; the latter methylation marks are removed by the H3K27 demethylase Jumonji domain-containing 3 (Jmjd3).

Cytokines and chemokines are critical for immune cell communication and TME recruitment, and their promoters are often epigenetically regulated. For instance, the overexpression of HDAC11 inhibits IL-10 expression and induces inflammatory APCs that can prime naïve T cells and restore the responsiveness of tolerant CD4+ T cells; conversely, a lack of HDAC11 causes the impairment of antigen-specific T cell responses [87]. More recently, HDAC11 was described as an essential regulator of IL-10 levels in myeloid cells in MDSC expansion [88]. Chemokines such as CXCL9, CXCL10, and CXCL11, which recruit CD8+ T cells, have been associated with improved responses to ICI therapy and better overall patient survival [82]. Epigenetic regulation of chemokine production can establish an immune-suppressive TME. For instance, trimethylation at H3K27 represses the production of CXCL9 and CXCL10 in ovarian cancer, establishing an immune-suppressive TME [89], while DNMT1 is responsible for the decreased CXCL12 in osteosarcomas, resulting in reduced CTL recruitment at the cancer site [90].

These findings indicate that exploring epigenetic biomarkers to predict responses to immune checkpoint inhibitor therapy potentially holds significant promise in improving patient outcomes.

## 5. Currently Studied Epigenomic Biomarkers of ICI Response

In the previous paragraphs, we described the crosstalk between epigenetic alterations and the immune system in cancer. Since it is possible that epigenetic predisposition and immune response translate into a favorable tumourigenic environment and outcome, the search for and identification of epigenomic alterations could provide an approach to identify biomarkers of response to ICI treatment strategies. Moreover, epigenetic biomarkers such as DNA methylation are often more stable in fluids and formalin-fixed, paraffin-embedded (FFPE) biospecimens as compared to mRNAs [35].

### 5.1. DNA Methylation and Epigenomic Signatures

DNA methylation plays an important role in modulating gene activity and gene silencing [91], as well as maintaining genomic stability. DNA methylation also facilitates genomic imprinting, X-chromosome inactivation, chromosome stabilization, and the repression of transposable elements [92,93,94]. However, cancer cells often exhibit global hypomethylation and promoter-specific hypermethylation of tumour suppressor genes during tumourigenesis, which is associated with gene silencing [95]. For example, the promoter of tumour suppressor gene *CDKN2A*, encoding p16^INK4a^, has been shown to be hypermethylated in metastatic melanoma, leading to p16 silencing [96]. Furthermore, methylation within the gene itself can induce mutational events [95]. Thus, sites of CpG DNA hypermethylation or hypomethylation in cancer [97] could be potential epigenetic signatures or biomarkers for evaluating the prognosis, diagnosis, or response to treatment in different types of cancer [92].

The comprehensive profiling of DNA methylation can be achieved using either microarrays (e.g., Infinium HumanMethylation450 BeadChip and Illumina MethylationEPIC BeadChip) or next generation sequencing (e.g., WGBS and RRBS). The MethylationEPIC BeadChip is capable of quantitatively analyzing the methylation levels at over 850,000 methylation sites across the genome with single-nucleotide resolution. This high-throughput approach provides comprehensive coverage of the methylome and offers the advantage of not being restricted to fresh tissue samples; it can also be effectively applied to formalin-fixed, paraffin-embedded (FFPE) tissue specimens. This capability is particularly advantageous for retrospective studies or when access to fresh tissue samples is limited.

Filipski et al. (2021) [98] used the Illumina MethylationEPIC BeadChip technology to investigate methylation signatures across the genomes of 61 stage IV melanoma patients who were treated with anti-PD-1-ICI during the course of their disease, along with Illumina 450 K methylation bead chip array data from a further 396 melanoma patients (stages I–IV, skin, soft tissue, central nervous system, peripheral, non-central nervous system organs, and lymph nodes) from The Cancer Genome Atlas (TCGA). This study performed a reference-free latent methylation components (LMC)-based DNA methylation data analysis technique. The authors showed that LMC proportion-based clustering in ICI-treated melanomas could predict durable long-term outcomes from ICI therapy. Moreover, since genome-wide methylation undergoes slower and sustained transformations within a dynamic tumour microenvironment, DNA methylation is thought to denote comparatively more stable signatures. This study also performed the deconvolution of DNA methylation data to identify immune cell methylation patterns that may serve as reliable biomarkers for the prediction of a successful ICI therapy response. However, this study was associated with several limitations, probably the most important of which was that the cutaneous melanoma tissues were collected after the ICI treatment had started, and so the tissues used for analysis had very likely undergone molecular alterations in response to the treatment. These molecular alterations could represent a confounding factor towards the identification of a biomarker for predicting the response to ICI therapy prior to starting treatment.

In another study, Ressler et al. [99] addressed this limitation by performing a similar approach to identify a set of CpG sites using pre-treated samples from metastatic melanoma patients to predict the response to ICI therapy. This study identified specific DNA methylation signatures, which revealed three distinct clusters based on the 500 most differentially methylated genes. These clusters allowed for the identification of responders (cluster 1 and cluster 2) from non-responders (cluster 3), and the findings from this study underscored the potential of DNA methylation profiling as an efficient predictive tool in the context of immunotherapy for metastatic melanoma.

Another group [100] conducted single methylation analysis of the CTLA-4 promoter using samples from 50 patients with metastasized malignant melanoma who were treated with anti-PD-1/CTLA-4 therapy. These authors used a methylation-specific quantitative real-time PCR technique. The findings revealed a significant correlation between low CTLA-4 methylation levels and both the response to therapy and overall survival.

### 5.2. Non-Coding RNAs

The ENCODE database reveals that the majority (~76%) of the human genome is transcribed, while only 2–3% of the genome consists of protein-coding genes; the remaining transcribed sequences comprise non-coding RNAs (ncRNAs) such as microRNAs (miRNA), small RNAs, PIWI-interacting RNAs, and various classes of long non-coding RNAs (lncRNAs). These ncRNAs are not only involved in the regulation of the transcriptional activities of single genes but also of entire transcriptional programs [33] as well as the cell cycle, apoptosis, and differentiation through acting as signals, scaffolds, molecular decoys, and sponges [34]. Malfunctions of ncRNAs could be involved in cancer progression, tumour growth, metastasis, and resistance to therapy by controlling the downregulation or upregulation of numerous genes [35], and indeed, ncRNAs are frequently altered in cancer tissues and are involved in innate and adaptive immunity in cancer. For example, lncRNAs interact with several immune microenvironment components, such as nuclear factor (NF)-κB, in the case of NF-κB-interacting lncRNA (*NKILA*), in tumour-specific cytotoxic T-lymphocytes (CTLs), or in tumour cells. Another example is a hypoxia-inducible factor 1α-stabilizing lncRNA in tumour-associated macrophages that is responsible for poor prognosis [101,102].

Yu et al. (2020) [103] identified novel lncRNA-based immune classes associated with cancer immunotherapy, and they recommended that immunotherapy would be more beneficial for patients in the active immune group. A cohort of 419 cancer patients from the TCGA (IMvigor210 trial cohort) was used to predict the association between lncRNAs and ICI therapy. Patients were grouped into four different classes such as “immune active”, “immune exclusion”, “immune dysfunctional”, and “immune desert” based on the presence of CTLs and lncRNA signatures. Patients with low lncRNA scores had longer survival compared to patients with high lncRNA scores. The “immune dysfunctional” class showed that dysfunctional lncRNAs were associated with closed interactions and, ultimately, immune escape, while, for example, *NKILA* expression involved an interaction with the NF-κB pathway, promoting an immunosuppressive microenvironment. This study therefore identified the potential of non-coding RNAs (particularly lncRNAs) as biomarkers for immunotherapy.

Chromatin modifications are associated with altered coding or non-coding RNA expression but aside from EZH2 and ARID2, relatively few chromatin modifiers have been investigated as biomarkers of ICI response in melanoma to date [97,98,99].

### 5.3. RNA Methylation

The most common post-transcriptional mRNA modification, N6-methyladenosine (m6A), regulates RNA splicing, nuclear export, stability, translation, DNA damage repair, the initiation of miRNA biogenesis, and immunogenicity. These processes affect cellular differentiation, embryonic development, spermatogenesis, sex determination, learning and memory, the immune response, and the occurrence and development of cancer [104]. RNA methylation is a biologically reversible process [36], occurring mostly in the mRNAs responsible for immune regulation. Moreover, RNA methylation influences immunogenicity and innate immune components and regulates tumour immunity, making it a potential candidate as a predictive biomarker for immunotherapy response. For example, by affecting inhibitor proteins in Tregs, m6A-modified mRNAs were found to help maintain the inhibitory function of Tregs [105]; without m6A modification, the Tregs lost their ability to inhibit T-cell proliferation [105].

In melanoma, m6A-marked mRNAs regulate neoantigen-specific immunity through YTH N6-Methyladenosine RNA Binding Protein 1 (YTHDF1) present in DCs. The binding of YTHDF1 to these transcripts increased the translation of lysosomal cathepsins in DCs, and the inhibition of cathepsins markedly enhanced cross-presentation by wild-type DCs. Therefore m6A plays an important role in the efficacy of tumour immunotherapy [39]. The loss of YTHDF1 was shown to increase neoantigen-specific CD8+ T cells and enhance the anti-tumour response of anti-PD1 therapy [106].

The inactivation of m6A regulators is associated with cancer metastasis in the liver, colon, kidney, and pancreas [107]. In addition, hypoxic conditions in breast cancer induce m6A demethylation and stabilize pluripotency factor NANOG, thereby promoting breast cancer stem cell phenotypes [108].

## 6. Limitations and Future Directions

Several studies have been carried out over the past few years to stratify patients as responders and non-responders to ICI therapy and to facilitate personalized medicine approaches in melanoma patients [98,99,100]. Understanding the dynamic nature of biomarkers in relation to ICI therapy response patterns could provide novel insights into overcoming resistance and tailoring treatment strategies for melanoma. Dynamic events occurring in the TME underpin the dynamic nature of predictive biomarkers, for which greater understanding is needed.

The TME plays a vital role in driving melanoma cells to switch their phenotype. Within the same melanoma tumour bed, melanoma can coexist in a range of phenotypic states; some cells may be differentiated and reflect the specialized function of the cell of origin [109]. A proportion of tumour cells may be actively cycling and, thus, fuel tumour growth, and a third class of cells may be invasive, some of which may have the potential to seed new metastases. Finally, dormant cells may lie quiescent for many years before their reactivation, when they may initiate a new tumour (i.e., metastatic lesion) or give rise to relapse after an apparently successful therapy [109,110]. Simultaneously, the immune components in the TME can also adapt to extrinsic stimuli, based on oxygen tension, glucose availability, or oxidoreduction pathways [111], leading to reprogramming of the TME [112].

The phenotypic status of melanoma cells in the melanoma tumour bed is influenced by and can influence the TME through interactions that involve both local and systemic effects. For example, local interactions through the regulation of melanin expression [113,114] result in the secretion of melanin into the TME, which can inhibit immune cell function, and lead to formation of cancer-associated fibroblasts, contributing to melanoma progression and aggressiveness [115]. The expression of melanin is characteristic of relatively differentiated melanoma cells, but ultimately, it promotes tumour progression. At a systemic level, TME interactions can occur through the secretion of neuroendocrine hormones from tumour cells [116]. For example, alpha melanocyte stimulating hormone (α-MSH) is a molecule highly expressed by melanoma cells, with autoregulatory effects mediated through its binding to melanocortin type 1 receptor (MC1R) [117]. Ultimately, neuroendocrine factors, as well as other extrinsic environmental factors including ultraviolet radiation [118], can influence the immune system, which may then impact melanoma progression.

To address our limited understanding of the TME, it is important to determine how different melanoma phenotypic states are initiated and maintained, how they influence tumour progression, and whether they exhibit any unique therapeutic vulnerabilities. Multi-faceted lines of enquiry in the future are likely to impact the prediction of ICI immunotherapy response in melanoma patients. More research is needed to better understand the implicit tumour heterogeneity in the TME. With further research, biomarkers present in the TME could significantly correlate with outcomes of melanoma ICI immunotherapy response. Investigating biomarkers in individual cells in the TME could further help in identifying aggressive emerging tumour cell subpopulations [119]. While initially rare, these aggressive melanoma cells could expand significantly. For this sort of approach, methodologies like single-cell sequencing [119], including single-cell DNA methylation sequencing, would be useful.

Ultimately, an integrated approach to evaluate both genomic and epigenomic biomarkers simultaneously, whereby an optimal combination of genomic and epigenomic biomarkers may improve the precision of ICI response prediction, could generate the most useful biomarker panels. However, irrespective of the development of genomic, epigenomic, or integrated genomic/epigenomic panels, the findings derived from biomarker studies should be validated in larger patient cohorts to ensure the development of the most robust biomarker panels that are both sensitive and accurate. Furthermore, mechanistic underpinnings of biomarkers that are eventually chosen to undergo development should be explored to enhance their clinical utility.

## 7. Conclusions

In summary, the most promising candidate predictive biomarkers for ICI response have not yet been identified. In this review, we outlined the published biomarkers for ICI therapy response, with a focus on genomic and epigenomic markers. This review highlights knowledge gaps in the potential identification of candidate biomarkers, which could be addressed in future research. The key reason for the limitations associated with currently available biomarkers is an absence of a proper understanding of the complex network of interactions of the TME that influence the efficacy of ICI response. The lack of an effective predictive biomarker impacts a significant fraction of patients who experience innate and acquired resistance followed by hyper-progression.

Despite evidence that epigenetic drugs like Decitabine, when used in combination with ICI therapies, lead to improved cancer patient outcomes, and also the potential promise regarding epigenetic regulation (involving both DNA and RNA modifications) for reprogramming events occurring during tumour immune evasion, few studies to date have reported the identification of either chromatin-associated or CpG-site- or m6A mRNA-specific epigenetic biomarkers of ICI response in human melanoma patients [120,121]. In this regard, DNA methylation stands out as a putative mechanism for the maintenance of the exhaustion of immune cells during ICI therapy. Detailed investigations into epigenetic regulators and their association with the clinical outcomes of ICIs in future work could reveal new biomarkers, while additionally, a greater understanding of the mechanisms of action of ICI therapy would also support the identification of new predictive biomarkers.

## Figures and Tables

**Table 1:** Biomarkers for predicting the response to immunotherapy treatment in metastatic melanoma patients.

Biomarkers	Mechanistic Insights	Ref.
Genomic Biomarkers		
PD-L1 expression in IHC	Epithelial cells can be induced to express PD-L1 in response to inflammatory cytokines, such as interferon-gamma, thus protecting these cells at sites of immune activation. PD-L1 may be expressed on tumour cells as well as inflammatory cells. The binding of PD-L1 with PD-1 or CD80 downregulates the response of activated T cells by inhibiting T-cell proliferation, cytokine production, and cytolytic activity, leading to the functional inactivation or exhaustion of T cells. Higher PD-L1 expression is often linked to better responses to ICI therapy. However, a lack of PD-L1 expression does not necessarily exclude the possibility of a response. Thus, the effectiveness of IHC detection of PD-L1, as a predictive biomarker in melanoma, is limited.	[15,16]
TMB	The evaluation of TMB is based on the hypothesis that a high number of mutations in exonic regions will lead to an increase in neoantigen production, which could then be recognized by CD8+ T cells, resulting in improved immune responses. Several variables may affect TMB determination: the depth of sequencing, length of sequencing reads, type of fixative agent, and fixation time, the latter of which influences the degree of formaldehyde-fixed, paraffin-embedded (FFPE), deamination-induced artifacts, all of which impact the analysis of TMB. In addition, a low tumour purity resulting from sampling errors may lead to reduced TMB assay sensitivity.	[26,27]
Neoantigen	Tumour types with a high TMB are theoretically often associated with a high neoantigen load. This is because a high TMB enhances the formation and presentation of immune neoantigens, leading to effective anti-tumour immune responses. It is speculated that tumours with a higher mutation burden possess more tumour-specific neoantigens, which in turn stimulates an increase in TILs due to the overexpression of immune checkpoint modulators such as PD-1 or PD-L1. However, TMB is not equivalent to a neoantigen load. One study found that half of oncogenic mutations did not result in neoantigens, indicating that TMB alone is not a reliable surrogate marker of immunogenic neoantigens.	[28,29,30]
dMMR and MSI-H	Tumours with mismatch repair deficiency (dMMR), either due to an inherited mutation or sporadic mutation, have a defect in one of the MMR genes (*MLH1*, *PMS2*, *MSH2*, or *MSH6*), resulting in the failure to repair errors in DNA replication. These errors are particularly prevalent in regions of repetitive DNA sequences known as microsatellites, resulting in high levels of microsatellite instability (MSI-H).	[31]
Epigenomic Biomarkers		

DNA methylation	Aberrant DNA methylation can alter the chromatin structure and gene transcription without altering the DNA sequence. Recent work revealed that DNA methylation affects tumourigenesis by regulating the tumour microenvironment.	[32]
Non-coding RNAs	Non-coding RNAs (ncRNAs) are involved in the regulation of the transcriptional activities of single genes, transcriptional programs, as well as the cell cycle, apoptosis, and differentiation. Malfunctions of ncRNAs could be involved in cancer progression, tumour growth, metastasis, and resistance to therapy by controlling the downregulation or upregulation of numerous genes. In general, ncRNAs are frequently altered in cancer tissues and are involved in innate and adaptive immunity in cancer.	[33,34,35]
RNA methylation	RNA methylation is a biologically reversible process and has been found to occur frequently in the mRNAs responsible for immune regulation. Moreover, RNA methylation influences immunogenicity, innate immune components, and regulates tumour immunity, making it a potential candidate as a predictive biomarker for ICI immunotherapy response.	[36]

## Data Availability

No new data were generated in this work.

## References

[1] Passarelli A., Mannavola F., Stucci L.S., Tucci M., Silvestris F. (2017). Immune system and melanoma biology: A balance between immunosurveillance and immune escape. Oncotarget.

[2] Gong J., Chehrazi-Raffle A., Reddi S., Salgia R. (2018). Development of PD-1 and PD-L1 inhibitors as a form of cancer immunotherapy: A comprehensive review of registration trials and future considerations. J. Immunother. Cancer.

[3] Vaddepally R.K., Kharel P., Pandey R., Garje R., Chandra A.B. (2020). Review of Indications of FDA-Approved Immune Checkpoint Inhibitors per NCCN Guidelines with the Level of Evidence. Cancers.

[4] Grigg C., Rizvi N.A. (2016). PD-L1 biomarker testing for non-small cell lung cancer: Truth or fiction?. J. Immunother. Cancer.

[5] Marconcini R., Spagnolo F., Stucci L.S., Ribero S., Marra E., Rosa F., Picasso V., Di Guardo L., Cimminiello C., Cavalieri S. (2018). Current status and perspectives in immunotherapy for metastatic melanoma. Oncotarget.

[6] Sharpe A.H., Pauken K.E. (2018). The diverse functions of the PD1 inhibitory pathway. Nat. Rev. Immunol..

[7] Chatterjee A., Rodger E.J., Ahn A., Stockwell P.A., Parry M., Motwani J., Gallagher S.J., Shklovskaya E., Tiffen J., Eccles M.R. (2018). Marked Global DNA Hypomethylation Is Associated with Constitutive PD-L1 Expression in Melanoma. iScience.

[8] Hossain S.M., Lynch-Sutherland C.F., Chatterjee A., Macaulay E.C., Eccles M.R. (2021). Can Immune Suppression and Epigenome Regulation in Placenta Offer Novel Insights into Cancer Immune Evasion and Immunotherapy Resistance?. Epigenomes.

[9] Koppolu V., Rekha Vasigala V.K. (2018). Checkpoint immunotherapy by nivolumab for treatment of metastatic melanoma. J. Cancer Res. Ther..

[10] Ventola C.L. (2017). Cancer Immunotherapy, Part 3: Challenges and Future Trends. P T.

[11] Jessurun C.A.C., Vos J.A.M., Limpens J., Luiten R.M. (2017). Biomarkers for Response of Melanoma Patients to Immune Checkpoint Inhibitors: A Systematic Review. Front. Oncol..

[12] Rotte A. (2019). Combination of CTLA-4 and PD-1 blockers for treatment of cancer. J. Exp. Clin. Cancer Res..

[13] Ankeny J.S., Labadie B., Luke J., Hsueh E., Messina J., Zager J.S. (2018). Review of diagnostic, prognostic, and predictive biomarkers in melanoma. Clin. Exp. Metastasis.

[14] Maher N.G., Vergara I.A., Long G.V., Scolyer R.A. (2024). Prognostic and predictive biomarkers in melanoma. Pathology.

[15] Paver E.C., Cooper W.A., Colebatch A.J., Ferguson P.M., Hill S.K., Lum T., Shin J.S., O’Toole S., Anderson L., Scolyer R.A. (2021). Programmed death ligand-1 (PD-L1) as a predictive marker for immunotherapy in solid tumours: A guide to immunohistochemistry implementation and interpretation. Pathology.

[16] Lantuejoul S., Sound-Tsao M., Cooper W.A., Girard N., Hirsch F.R., Roden A.C., Lopez-Rios F., Jain D., Chou T.Y., Motoi N. (2020). PD-L1 Testing for Lung Cancer in 2019: Perspective From the IASLC Pathology Committee. J. Thorac. Oncol..

[17] Cha J.H., Chan L.C., Li C.W., Hsu J.L., Hung M.C. (2019). Mechanisms Controlling PD-L1 Expression in Cancer. Mol. Cell.

[18] Jiang Y., Chen M., Nie H., Yuan Y. (2019). PD-1 and PD-L1 in cancer immunotherapy: Clinical implications and future considerations. Hum. Vaccines Immunother..

[19] Patel S.P., Kurzrock R. (2015). PD-L1 Expression as a Predictive Biomarker in Cancer Immunotherapy. Mol. Cancer Ther..

[20] Maleki Vareki S., Garrigos C., Duran I. (2017). Biomarkers of response to PD-1/PD-L1 inhibition. Crit. Rev. Oncol./Hematol..

[21] Madore J., Vilain R.E., Menzies A.M., Kakavand H., Wilmott J.S., Hyman J., Yearley J.H., Kefford R.F., Thompson J.F., Long G.V. (2015). PD-L1 expression in melanoma shows marked heterogeneity within and between patients: Implications for anti-PD-1/PD-L1 clinical trials. Pigment. Cell Melanoma Res..

[22] Topalian S.L., Taube J.M., Anders R.A., Pardoll D.M. (2016). Mechanism-driven biomarkers to guide immune checkpoint blockade in cancer therapy. Nat. Rev. Cancer.

[23] Kamel H.F.M., Al-Amodi H. (2017). Exploitation of Gene Expression and Cancer Biomarkers in Paving the Path to Era of Personalized Medicine. Genom. Proteom. Bioinform..

[24] Goossens N., Nakagawa S., Sun X., Hoshida Y. (2015). Cancer biomarker discovery and validation. Transl. Cancer Res..

[25] Roccuzzo G., Bongiovanni E., Tonella L., Pala V., Marchisio S., Ricci A., Senetta R., Bertero L., Ribero S., Berrino E. (2024). Emerging prognostic biomarkers in advanced cutaneous melanoma: A literature update. Expert. Rev. Mol. Diagn..

[26] Fumet J.D., Truntzer C., Yarchoan M., Ghiringhelli F. (2020). Tumour mutational burden as a biomarker for immunotherapy: Current data and emerging concepts. Eur. J. Cancer.

[27] Rizvi N.A., Hellmann M.D., Snyder A., Kvistborg P., Makarov V., Havel J.J., Lee W., Yuan J., Wong P., Ho T.S. (2015). Cancer immunology. Mutational landscape determines sensitivity to PD-1 blockade in non-small cell lung cancer. Science.

[28] Maleki Vareki S. (2018). High and low mutational burden tumors versus immunologically hot and cold tumors and response to immune checkpoint inhibitors. J. Immunother. Cancer.

[29] Gong L., He R., Xu Y., Luo T., Jin K., Yuan W., Zheng Z., Liu L., Liang Z., Li A. (2021). Neoantigen load as a prognostic and predictive marker for stage II/III non-small cell lung cancer in Chinese patients. Thorac. Cancer.

[30] Zou X.L., Li X.B., Ke H., Zhang G.Y., Tang Q., Yuan J., Zhou C.J., Zhang J.L., Zhang R., Chen W.Y. (2021). Prognostic Value of Neoantigen Load in Immune Checkpoint Inhibitor Therapy for Cancer. Front. Immunol..

[31] Maio M., Ascierto P.A., Manzyuk L., Motola-Kuba D., Penel N., Cassier P.A., Bariani G.M., De Jesus Acosta A., Doi T., Longo F. (2022). Pembrolizumab in microsatellite instability high or mismatch repair deficient cancers: Updated analysis from the phase II KEYNOTE-158 study. Ann. Oncol..

[32] Xue G., Cui Z.J., Zhou X.H., Zhu Y.X., Chen Y., Liang F.J., Tang D.N., Huang B.Y., Zhang H.Y., Hu Z.H. (2019). DNA Methylation Biomarkers Predict Objective Responses to PD-1/PD-L1 Inhibition Blockade. Front. Genet..

[33] Kugel J.F., Goodrich J.A. (2012). Non-coding RNAs: Key regulators of mammalian transcription. Trends Biochem. Sci..

[34] Yang X., Liu M., Li M., Zhang S., Hiju H., Sun J., Mao Z., Zheng M., Feng B. (2020). Epigenetic modulations of noncoding RNA: A novel dimension of Cancer biology. Mol. Cancer.

[35] García-Giménez J.L., Ushijima T., Tollefsbol T.O. (2016). Chapter 1—Epigenetic Biomarkers: New Findings, Perspectives, and Future Directions in Diagnostics. Epigenetic Biomarkers and Diagnostics.

[36] Chen X.Y., Zhang J., Zhu J.S. (2019). The role of m(6)A RNA methylation in human cancer. Mol. Cancer.

[37] Schwitalle Y., Kloor M., Eiermann S., Linnebacher M., Kienle P., Knaebel H.P., Tariverdian M., Benner A., von Knebel Doeberitz M. (2008). Immune response against frameshift-induced neopeptides in HNPCC patients and healthy HNPCC mutation carriers. Gastroenterology.

[38] Yamashita H., Nakayama K., Ishikawa M., Nakamura K., Ishibashi T., Sanuki K., Ono R., Sasamori H., Minamoto T., Iida K. (2018). Microsatellite instability is a biomarker for immune checkpoint inhibitors in endometrial cancer. Oncotarget.

[39] Yarchoan M., Hopkins A., Jaffee E.M. (2017). Tumor Mutational Burden and Response Rate to PD-1 Inhibition. N. Engl. J. Med..

[40] McGranahan N., Furness A.J., Rosenthal R., Ramskov S., Lyngaa R., Saini S.K., Jamal-Hanjani M., Wilson G.A., Birkbak N.J., Hiley C.T. (2016). Clonal neoantigens elicit T cell immunoreactivity and sensitivity to immune checkpoint blockade. Science.

[41] Miao D., Margolis C.A., Gao W., Voss M.H., Li W., Martini D.J., Norton C., Bosse D., Wankowicz S.M., Cullen D. (2018). Genomic correlates of response to immune checkpoint therapies in clear cell renal cell carcinoma. Science.

[42] Miao D., Margolis C.A., Vokes N.I., Liu D., Taylor-Weiner A., Wankowicz S.M., Adeegbe D., Keliher D., Schilling B., Tracy A. (2018). Genomic correlates of response to immune checkpoint blockade in microsatellite-stable solid tumors. Nat. Genet..

[43] Gao J., Shi L.Z., Zhao H., Chen J., Xiong L., He Q., Chen T., Roszik J., Bernatchez C., Woodman S.E. (2016). Loss of IFN-gamma Pathway Genes in Tumor Cells as a Mechanism of Resistance to Anti-CTLA-4 Therapy. Cell.

[44] Zaretsky J.M., Garcia-Diaz A., Shin D.S., Escuin-Ordinas H., Hugo W., Hu-Lieskovan S., Torrejon D.Y., Abril-Rodriguez G., Sandoval S., Barthly L. (2016). Mutations Associated with Acquired Resistance to PD-1 Blockade in Melanoma. N. Engl. J. Med..

[45] Chowell D., Krishna C., Pierini F., Makarov V., Rizvi N.A., Kuo F., Morris L.G.T., Riaz N., Lenz T.L., Chan T.A. (2019). Evolutionary divergence of HLA class I genotype impacts efficacy of cancer immunotherapy. Nat. Med..

[46] Snyder A., Makarov V., Merghoub T., Yuan J., Zaretsky J.M., Desrichard A., Walsh L.A., Postow M.A., Wong P., Ho T.S. (2014). Genetic basis for clinical response to CTLA-4 blockade in melanoma. N. Engl. J. Med..

[47] Brown S.D., Warren R.L., Gibb E.A., Martin S.D., Spinelli J.J., Nelson B.H., Holt R.A. (2014). Neo-antigens predicted by tumor genome meta-analysis correlate with increased patient survival. Genome Res..

[48] Mauriello A., Zeuli R., Cavalluzzo B., Petrizzo A., Tornesello M.L., Buonaguro F.M., Ceccarelli M., Tagliamonte M., Buonaguro L. (2019). High Somatic Mutation and Neoantigen Burden Do Not Correlate with Decreased Progression-Free Survival in HCC Patients not Undergoing Immunotherapy. Cancers.

[49] Karpanen T., Olweus J. (2017). The Potential of Donor T-Cell Repertoires in Neoantigen-Targeted Cancer Immunotherapy. Front. Immunol..

[50] Koster B.D., de Gruijl T.D., van den Eertwegh A.J. (2015). Recent developments and future challenges in immune checkpoint inhibitory cancer treatment. Curr. Opin. Oncol..

[51] Koyama S., Akbay E.A., Li Y.Y., Herter-Sprie G.S., Buczkowski K.A., Richards W.G., Gandhi L., Redig A.J., Rodig S.J., Asahina H. (2016). Adaptive resistance to therapeutic PD-1 blockade is associated with upregulation of alternative immune checkpoints. Nat. Commun..

[52] Maeurer M.J., Gollin S.M., Storkus W.J., Swaney W., Karbach J., Martin D., Castelli C., Salter R., Knuth A., Lotze M.T. (1996). Tumor escape from immune recognition: Loss of HLA-A2 melanoma cell surface expression is associated with a complex rearrangement of the short arm of chromosome 6. Clin. Cancer Res..

[53] Gettinger S.N., Horn L., Gandhi L., Spigel D.R., Antonia S.J., Rizvi N.A., Powderly J.D., Heist R.S., Carvajal R.D., Jackman D.M. (2015). Overall Survival and Long-Term Safety of Nivolumab (Anti-Programmed Death 1 Antibody, BMS-936558, ONO-4538) in Patients With Previously Treated Advanced Non-Small-Cell Lung Cancer. J. Clin. Oncol..

[54] Trabucco S.E., Gowen K., Maund S.L., Sanford E., Fabrizio D.A., Hall M.J., Yakirevich E., Gregg J.P., Stephens P.J., Frampton G.M. (2019). A Novel Next-Generation Sequencing Approach to Detecting Microsatellite Instability and Pan-Tumor Characterization of 1000 Microsatellite Instability-High Cases in 67,000 Patient Samples. J. Mol. Diagn..

[55] Modrich P. (2006). Mechanisms in eukaryotic mismatch repair. J. Biol. Chem..

[56] Zhao P., Li L., Jiang X., Li Q. (2019). Mismatch repair deficiency/microsatellite instability-high as a predictor for anti-PD-1/PD-L1 immunotherapy efficacy. J. Hematol. Oncol..

[57] Iyer R.R., Pluciennik A., Burdett V., Modrich P.L. (2006). DNA mismatch repair: Functions and mechanisms. Chem. Rev..

[58] Negureanu L., Salsbury F.R. (2012). The molecular origin of the MMR-dependent apoptosis pathway from dynamics analysis of MutSalpha-DNA complexes. J. Biomol. Struct. Dyn..

[59] Lynch H.T., Jascur T., Lanspa S., Boland C.R. (2010). Making sense of missense in Lynch syndrome: The clinical perspective. Cancer Prev. Res..

[60] Boland C.R., Goel A. (2010). Microsatellite instability in colorectal cancer. Gastroenterology.

[61] Beggs A.D., Domingo E., Abulafi M., Hodgson S.V., Tomlinson I.P. (2013). A study of genomic instability in early preneoplastic colonic lesions. Oncogene.

[62] Funkhouser W.K., Lubin I.M., Monzon F.A., Zehnbauer B.A., Evans J.P., Ogino S., Nowak J.A. (2012). Relevance, pathogenesis, and testing algorithm for mismatch repair-defective colorectal carcinomas: A report of the association for molecular pathology. J. Mol. Diagn..

[63] Timmermann B., Kerick M., Roehr C., Fischer A., Isau M., Boerno S.T., Wunderlich A., Barmeyer C., Seemann P., Koenig J. (2010). Somatic mutation profiles of MSI and MSS colorectal cancer identified by whole exome next generation sequencing and bioinformatics analysis. PLoS ONE.

[64] Hsieh P., Yamane K. (2008). DNA mismatch repair: Molecular mechanism, cancer, and ageing. Mech. Ageing Dev..

[65] Llosa N.J., Cruise M., Tam A., Wicks E.C., Hechenbleikner E.M., Taube J.M., Blosser R.L., Fan H., Wang H., Luber B.S. (2015). The vigorous immune microenvironment of microsatellite instable colon cancer is balanced by multiple counter-inhibitory checkpoints. Cancer Discov..

[66] Saeterdal I., Bjørheim J., Lislerud K., Gjertsen M.K., Bukholm I.K., Olsen O.C., Nesland J.M., Eriksen J.A., Møller M., Lindblom A. (2001). Frameshift-mutation-derived peptides as tumor-specific antigens in inherited and spontaneous colorectal cancer. Proc. Natl. Acad. Sci. USA.

[67] Boissière-Michot F., Lazennec G., Frugier H., Jarlier M., Roca L., Duffour J., Du Paty E., Laune D., Blanchard F., Le Pessot F. (2014). Characterization of an adaptive immune response in microsatellite-instable colorectal cancer. Oncoimmunology.

[68] Cicek M.S., Lindor N.M., Gallinger S., Bapat B., Hopper J.L., Jenkins M.A., Young J., Buchanan D., Walsh M.D., Le Marchand L. (2011). Quality assessment and correlation of microsatellite instability and immunohistochemical markers among population- and clinic-based colorectal tumors results from the Colon Cancer Family Registry. J. Mol. Diagn..

[69] Overman M.J., McDermott R., Leach J.L., Lonardi S., Lenz H.J., Morse M.A., Desai J., Hill A., Axelson M., Moss R.A. (2017). Nivolumab in patients with metastatic DNA mismatch repair-deficient or microsatellite instability-high colorectal cancer (CheckMate 142): An open-label, multicentre, phase 2 study. Lancet Oncol..

[70] Sun H., Huang B., Cao J., Yan Q., Yin M. (2022). Editorial: Epigenetic Regulation and Tumor Immunotherapy. Front. Oncol..

[71] Sharma S., Kelly T.K., Jones P.A. (2010). Epigenetics in cancer. Carcinogenesis.

[72] Villanueva L., Álvarez-Errico D., Esteller M. (2020). The Contribution of Epigenetics to Cancer Immunotherapy. Trends Immunol..

[73] Ghoneim H.E., Fan Y., Moustaki A., Abdelsamed H.A., Dash P., Dogra P., Carter R., Awad W., Neale G., Thomas P.G. (2017). De Novo Epigenetic Programs Inhibit PD-1 Blockade-Mediated T Cell Rejuvenation. Cell.

[74] Pauken K.E., Sammons M.A., Odorizzi P.M., Manne S., Godec J., Khan O., Drake A.M., Chen Z., Sen D.R., Kurachi M. (2016). Epigenetic stability of exhausted T cells limits durability of reinvigoration by PD-1 blockade. Science.

[75] Crompton J.G., Narayanan M., Cuddapah S., Roychoudhuri R., Ji Y., Yang W., Patel S.J., Sukumar M., Palmer D.C., Peng W. (2016). Lineage relationship of CD8(+) T cell subsets is revealed by progressive changes in the epigenetic landscape. Cell. Mol. Immunol..

[76] Hackl H., Charoentong P., Finotello F., Trajanoski Z. (2016). Computational genomics tools for dissecting tumour-immune cell interactions. Nat. Rev. Genet..

[77] Chen D.S., Mellman I. (2013). Oncology meets immunology: The cancer-immunity cycle. Immunity.

[78] Tay R.E., Richardson E.K., Toh H.C. (2021). Revisiting the role of CD4(+) T cells in cancer immunotherapy-new insights into old paradigms. Cancer Gene Ther..

[79] Scharer C.D., Barwick B.G., Youngblood B.A., Ahmed R., Boss J.M. (2013). Global DNA methylation remodeling accompanies CD8 T cell effector function. J. Immunol..

[80] Lewis C.E., Pollard J.W. (2006). Distinct role of macrophages in different tumor microenvironments. Cancer Res..

[81] Lee H., Ferguson A.L., Quek C., Vergara I.A., Pires daSilva I., Allen R., Gide T.N., Conway J.W., Koufariotis L.T., Hayward N.K. (2023). Intratumoral CD16+ Macrophages Are Associated with Clinical Outcomes of Patients with Metastatic Melanoma Treated with Combination Anti-PD-1 and Anti-CTLA-4 Therapy. Clin. Cancer Res..

[82] House I.G., Savas P., Lai J., Chen A.X.Y., Oliver A.J., Teo Z.L., Todd K.L., Henderson M.A., Giuffrida L., Petley E.V. (2020). Macrophage-Derived CXCL9 and CXCL10 Are Required for Antitumor Immune Responses Following Immune Checkpoint Blockade. Clin. Cancer Res..

[83] Antoranz A., Van Herck Y., Bolognesi M.M., Lynch S.M., Rahman A., Gallagher W.M., Boecxstaens V., Marine J.C., Cattoretti G., van den Oord J.J. (2022). Mapping the Immune Landscape in Metastatic Melanoma Reveals Localized Cell-Cell Interactions That Predict Immunotherapy Response. Cancer Res..

[84] Hossain S.M., Gimenez G., Stockwell P.A., Tsai P., Print C.G., Rys J., Cybulska-Stopa B., Ratajska M., Harazin-Lechowska A., Almomani S. (2022). Innate immune checkpoint inhibitor resistance is associated with melanoma sub-types exhibiting invasive and de-differentiated gene expression signatures. Front. Immunol..

[85] Yang X., Wang X., Liu D., Yu L., Xue B., Shi H. (2014). Epigenetic regulation of macrophage polarization by DNA methyltransferase 3b. Mol. Endocrinol..

[86] Ishii M., Wen H., Corsa C.A., Liu T., Coelho A.L., Allen R.M., Carson W.F.t., Cavassani K.A., Li X., Lukacs N.W. (2009). Epigenetic regulation of the alternatively activated macrophage phenotype. Blood.

[87] Villagra A., Cheng F., Wang H.W., Suarez I., Glozak M., Maurin M., Nguyen D., Wright K.L., Atadja P.W., Bhalla K. (2009). The histone deacetylase HDAC11 regulates the expression of interleukin 10 and immune tolerance. Nat. Immunol..

[88] Sahakian E., Powers J.J., Chen J., Deng S.L., Cheng F., Distler A., Woods D.M., Rock-Klotz J., Sodre A.L., Youn J.I. (2015). Histone deacetylase 11: A novel epigenetic regulator of myeloid derived suppressor cell expansion and function. Mol. Immunol..

[89] Peng D., Kryczek I., Nagarsheth N., Zhao L., Wei S., Wang W., Sun Y., Zhao E., Vatan L., Szeliga W. (2015). Epigenetic silencing of TH1-type chemokines shapes tumour immunity and immunotherapy. Nature.

[90] Li B., Wang Z., Wu H., Xue M., Lin P., Wang S., Lin N., Huang X., Pan W., Liu M. (2018). Epigenetic Regulation of CXCL12 Plays a Critical Role in Mediating Tumor Progression and the Immune Response In Osteosarcoma. Cancer Res..

[91] Papaiz D.D., Rius F.E., Ayub A.L.P., Origassa C.S., Gujar H., Pessoa D.O., Reis E.M., Nsengimana J., Newton-Bishop J., Mason C.E. (2022). Genes regulated by DNA methylation are involved in distinct phenotypes during melanoma progression and are prognostic factors for patients. Mol. Oncol..

[92] Micevic G., Theodosakis N., Bosenberg M. (2017). Aberrant DNA methylation in melanoma: Biomarker and therapeutic opportunities. Clin. Epigenetics.

[93] Huan T., Joehanes R., Song C., Peng F., Guo Y., Mendelson M., Yao C., Liu C., Ma J., Richard M. (2019). Genome-wide identification of DNA methylation QTLs in whole blood highlights pathways for cardiovascular disease. Nat. Commun..

[94] Schinke C., Mo Y., Yu Y., Amiri K., Sosman J., Greally J., Verma A. (2010). Aberrant DNA methylation in malignant melanoma. Melanoma Res..

[95] Wajed S.A., Laird P.W., DeMeester T.R. (2001). DNA methylation: An alternative pathway to cancer. Ann. Surg..

[96] Zhao R., Choi B.Y., Lee M.H., Bode A.M., Dong Z. (2016). Implications of Genetic and Epigenetic Alterations of CDKN2A (p16(INK4a)) in Cancer. EBioMedicine.

[97] Torano E.G., Petrus S., Fernandez A.F., Fraga M.F. (2012). Global DNA hypomethylation in cancer: Review of validated methods and clinical significance. Clin. Chem. Lab. Med..

[98] Filipski K., Scherer M., Zeiner K.N., Bucher A., Kleemann J., Jurmeister P., Hartung T.I., Meissner M., Plate K.H., Fenton T.R. (2021). DNA methylation-based prediction of response to immune checkpoint inhibition in metastatic melanoma. J. Immunother. Cancer.

[99] Ressler J.M., Tomasich E., Hatziioannou T., Ringl H., Heller G., Silmbrod R., Gottmann L., Starzer A.M., Zila N., Tschandl P. (2024). DNA Methylation Signatures Correlate with Response to Immune Checkpoint Inhibitors in Metastatic Melanoma. Target. Oncol..

[100] Goltz D., Gevensleben H., Vogt T.J., Dietrich J., Golletz C., Bootz F., Kristiansen G., Landsberg J., Dietrich D. (2018). CTLA4 methylation predicts response to anti-PD-1 and anti-CTLA-4 immunotherapy in melanoma patients. JCI Insight.

[101] Huang D., Chen J., Yang L., Ouyang Q., Li J., Lao L., Zhao J., Liu J., Lu Y., Xing Y. (2018). NKILA lncRNA promotes tumor immune evasion by sensitizing T cells to activation-induced cell death. Nat. Immunol..

[102] Chen F., Chen J., Yang L., Liu J., Zhang X., Zhang Y., Tu Q., Yin D., Lin D., Wong P.P. (2019). Extracellular vesicle-packaged HIF-1alpha-stabilizing lncRNA from tumour-associated macrophages regulates aerobic glycolysis of breast cancer cells. Nat. Cell Biol..

[103] Yu Y., Zhang W., Li A., Chen Y., Ou Q., He Z., Zhang Y., Liu R., Yao H., Song E. (2020). Association of Long Noncoding RNA Biomarkers With Clinical Immune Subtype and Prediction of Immunotherapy Response in Patients With Cancer. JAMA Netw. Open.

[104] Zhang M., Song J., Yuan W., Zhang W., Sun Z. (2021). Roles of RNA Methylation on Tumor Immunity and Clinical Implications. Front. Immunol..

[105] Tong J., Cao G., Zhang T., Sefik E., Amezcua Vesely M.C., Broughton J.P., Zhu S., Li H., Li B., Chen L. (2018). m^6^A mRNA methylation sustains Treg suppressive functions. Cell Res..

[106] Han D., Liu J., Chen C., Dong L., Liu Y., Chang R., Huang X., Liu Y., Wang J., Dougherty U. (2019). Anti-tumour immunity controlled through mRNA m^6^A methylation and YTHDF1 in dendritic cells. Nature.

[107] Liang Y., Zhang X., Ma C., Hu J. (2022). m^6^A Methylation Regulators Are Predictive Biomarkers for Tumour Metastasis in Prostate Cancer. Cancers.

[108] Zhang C., Samanta D., Lu H., Bullen J.W., Zhang H., Chen I., He X., Semenza G.L. (2016). Hypoxia induces the breast cancer stem cell phenotype by HIF-dependent and ALKBH5-mediated m⁶A-demethylation of NANOG mRNA. Proc. Natl. Acad. Sci. USA.

[109] Giancotti F.G. (2013). Mechanisms governing metastatic dormancy and reactivation. Cell.

[110] Sosa M.S., Bragado P., Aguirre-Ghiso J.A. (2014). Mechanisms of disseminated cancer cell dormancy: An awakening field. Nat. Rev. Cancer.

[111] Milotti E., Fredrich T., Chignola R., Rieger H. (2020). Oxygen in the Tumor Microenvironment: Mathematical and Numerical Modeling. Adv. Exp. Med. Biol..

[112] Reinfeld B.I., Madden M.Z., Wolf M.M., Chytil A., Bader J.E., Patterson A.R., Sugiura A., Cohen A.S., Ali A., Do B.T. (2021). Cell-programmed nutrient partitioning in the tumour microenvironment. Nature.

[113] Slominski A., Zmijewski M.A., Pawelek J. (2012). L-tyrosine and L-dihydroxyphenylalanine as hormone-like regulators of melanocyte functions. Pigment. Cell Melanoma Res..

[114] Slominski A., Tobin D.J., Shibahara S., Wortsman J. (2004). Melanin pigmentation in mammalian skin and its hormonal regulation. Physiol. Rev..

[115] Cabaço L.C., Tomás A., Pojo M., Barralm D.C. (2022). The Dark Side of Melanin Secretion in Cutaneous Melanoma Aggressiveness. Front. Oncol..

[116] Slominski R.M., Raman C., Chen J.Y., Slominski A.T. (2023). How cancer hijacks the body’s homeostasis through the neuroendocrine system. Trends Neurosci..

[117] Dall’Olmo L., Papa N., Surdo N.C., Marigo I., Mocellin S. (2023). Alpha-melanocyte stimulating hormone (α-MSH): Biology, clinical relevance and implication in melanoma. J. Transl. Med..

[118] Slominski R.M., Chen J.Y., Raman C., Slominski A.T. (2024). Photo-neuro-immuno-endocrinology: How the ultraviolet radiation regulates the body, brain, and immune system. Proc. Natl. Acad. Sci. USA.

[119] Niebel D., Fröhlich A., Zarbl R., Fietz S., de Vos L., Vogt T.J., Dietrich J., Sirokay J., Kuster P., Saavedra G. (2022). DNA methylation regulates TIGIT expression within the melanoma microenvironment, is prognostic for overall survival, and predicts progression-free survival in patients treated with anti-PD-1 immunotherapy. Clin. Epigenetics.

[120] Xu Y., Li P., Liu Y., Xin D., Lei W., Liang A., Han W., Qian W. (2022). Epi-immunotherapy for cancers: Rationales of epi-drugs in combination with immunotherapy and advances in clinical trials. Cancer Commun..

[121] Hogg S.J., Beavis P.A., Dawson M.A., Johnstone R.W. (2020). Targeting the epigenetic regulation of antitumour immunity. Nat. Rev. Drug Discov..

